# Multiplex Amplification Refractory Mutation System Polymerase Chain Reaction (ARMS-PCR) for diagnosis of natural infection with canine distemper virus

**DOI:** 10.1186/1743-422X-7-122

**Published:** 2010-06-10

**Authors:** Songkhla Chulakasian, Min-Shiuh Lee, Chi-Young Wang, Shyan-Song Chiou, Kuan-Hsun Lin, Fong-Yuan Lin, Tien-Huan Hsu, Min-Liang Wong, Tien-Jye Chang, Wei-Li Hsu

**Affiliations:** 1 Department of Veterinary Medicine, College of Veterinary Medicine, National Chung Hsing University, 250 Kou Kuang Road, Taichung 402, Taiwan; 2 Animal Health Research Institute, Council of Agriculture, 376 Chung-Cheng Road, Tamsui, Taipei 251, Taiwan; 3 Graduate Institute of Microbiology and Public Health, College of Veterinary Medicine, National Chung Hsing University, 250 Kou Kuang Road, Taichung 402, Taiwan

## Abstract

**Background:**

Canine distemper virus (CDV) is present worldwide and produces a lethal systemic infection of wild and domestic *Canidae*. Pre-existing antibodies acquired from vaccination or previous CDV infection might interfere the interpretation of a serologic diagnosis method. In addition, due to the high similarity of nucleic acid sequences between wild-type CDV and the new vaccine strain, current PCR derived methods cannot be applied for the definite confirmation of CD infection. Hence, it is worthy of developing a simple and rapid nucleotide-based assay for differentiation of wild-type CDV which is a cause of disease from attenuated CDVs after vaccination. High frequency variations have been found in the region spanning from the 3'-untranslated region (UTR) of the matrix (M) gene to the fusion (F) gene (designated M-F UTR) in a few CDV strains. To establish a differential diagnosis assay, an amplification refractory mutation analysis was established based on the highly variable region on M-F UTR and F regions.

**Results:**

Sequences of frequent polymorphisms were found scattered throughout the M-F UTR region; the identity of nucleic acid between local strains and vaccine strains ranged from 82.5% to 93.8%. A track of AAA residue located 35 nucleotides downstream from F gene start codon highly conserved in three vaccine strains were replaced with TGC in the local strains; that severed as target sequences for deign of discrimination primers. The method established in the present study successfully differentiated seven Taiwanese CDV field isolates, all belonging to the Asia-1 lineage, from vaccine strains.

**Conclusions:**

The method described herein would be useful for several clinical applications, such as confirmation of nature CDV infection, evaluation of vaccination status and verification of the circulating viral genotypes.

## Background

Canine distemper is a highly contagious disease caused by canine distemper virus (CDV), which belongs to the genus *Morbillivirus *of the family *Paramyxoviridae*. Although CDV primarily infects canids, infection of other terrestrial and aquatic carnivores has been reported [1-7]. CDV infection causes a systemic disease with severe immunosuppression involving primary replication of the virus in macrophages and lymphocytes of the respiratory tract, as well as in various lymphoid tissues [8].

The genome of CDV is approximately 15.7 kb in length and consists of a single-stranded, negative-sense RNA encoding the following eight viral proteins: two transcriptase-associated proteins (the phosphoprotein P and the large protein L) and the nucleocapsid protein (N) that encapsidates the viral RNA, a single envelope-associated matrix (M) protein and two glycoproteins: haemagglutinin/attachment protein (H) and a fusion protein (F) [9]. The F protein is responsible for viral fusion with host cells. The open reading frame of the F gene encodes 662 amino acids, which comprise a pre-signal peptide (Fsp), the F1 subunit and the F2 subunit; the latter two subunits are produced via post-translational proteolysis of the primary translation precursor product, designated pre-F0 [10,11].

CDVs worldwide could be clustered into six major genetic lineages; America, European, Asia-1, Asia-2, Arctic, and Vaccine [12-16]. Over the last five decades, CDV isolates from the latter lineage, such as Onderstepoort, and Snyder Hill, were applied in vaccine production and used as conventional distemper vaccines [17,18]. Recently, a new vaccine based on the contemporary vaccine strain (Vaccine X, GenBank: EU072198) has been used for immunisation. Sequence analysis, however, revealed that the contemporary strain used for Vaccine X is genetically distinct from the other CDVs in the vaccine lineage (used in conventional distemper vaccines).

Canine distemper is an incurable multisystemic viral disease that causes respiratory signs, gastrointestinal disorders, and progressive neurological signs. Prevention of CDV infection mainly relies on the use of live attenuated vaccines. Current routine serological tests detecting serum antibody titers are difficult to distinguish that animals have been vaccinated or late in infection as the modified live vaccines may result in a false positive in the first few weeks after immunisation. This rise the difficulties not only in the epidemic surveillance monitoring CDV outbreaks in domestic and wild animals, but also in the clinical diagnosis as a reference for treatment strategies, either continuing therapy or euthanasia. Recently, several molecular based assays have been established [12,15,19-21] to definitively clarify CDV infection. These molecular methods can only differentiate wild type and conventional vaccine strians. However, they are not able to identify the contemporary vaccine strain from the circulating wild type CDVs and thus it is possible that dogs vaccinated with the contemporary vaccine could be regarded as wild type CDV infection.

The goal of this study is to establish a simple and rapid assay for differentiating CDV of natural infection from that of vaccination which could be broadly adopted in countries where both conventional and contemporary distemper vaccines are commonly used in the vaccination program. Our previous report showed that there was a remarkable genetic diversity in the Fsp region among different CDV isolates [22], we further examined variation of Fsp and its upstream non-coding region (M-F untranslated region; M-F UTR) between circulating wild-type CDV and the vaccine strains in Taiwan. Toward this objective, CDVs from local isolates and three commonly used vaccines were sequenced and subjected to phylogenetic analysis. In addition, based on the determined divergent sequences, a multiplex ARMS-PCR system and enzyme recognition profile for the F gene and its upstream non-coding region were accordingly developed and successfully applied to the differentiation of vaccine strains and field isolates.

## Results

### Sequence and phylogenetic analysis

To determine the phylogenetic relationships among of CDV field and vaccine strains, considering the limited sequence information of F gene from other countries, phylogenetic analysis of H gene was conducted to determine the lineage relationship of various CDV strains. Initially, full-length H gene sequences of the seven local CDV were identified (GenBank: FJ705230 to FJ705239). Consistent with our previous report [23], the local strains originated from CDV-Asia-1 lineage. Furthermore, unlike vacc-Q and Vacc-N (Onderstepoort strain), Vacc-P was distinct, placed near strains of America (additional file 1).

**Figure 1:**
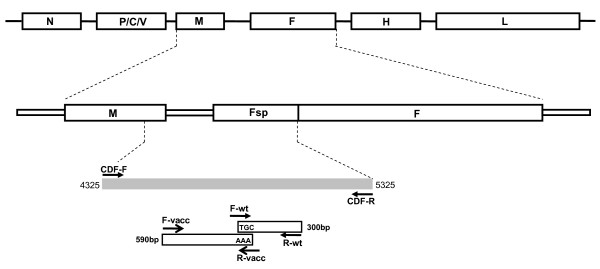
**Schematic illustration of the CDV genome and the locations of the primers used in this study**. The primer pairs CDF-F and CDF-R were designed for the first round amplification. Two inner primer sets F-vacc/R-vacc and F-wt/R-wt were simultaneously used for the second round multiplex ARMS-PCR. The F-wt and R-vacc were designed to differential amplification of field and vaccine strains, respectively. Arrows indicate the direction of primers.

By means of PCR with primer set CDV-F/R (the location was illustrated in Fig 1), sequences of F gene plus the upstream M-F intergenic region (nucleotides 4325-5325) were identified from seven CDV confirmed cases (namely TW1 to TW7) that were used to represent local strains (Asia-1 lineage) and three most commonly used commercial vaccines in Taiwan. These sequences were submitted to GenBank (GenBank: FJ694842 to FJ694848 for the field isolates and FJ694849, FJ694850 and FJ694851 for vaccines N, P and Q, respectively). Alignment of the nucleotide sequences using Clustal W demonstrated that the sequence identities among local isolates ranged from 96.8-100%, while those of the vaccine isolates were lower at 86.2-96.3% (Table 1). Interestingly, the nucleotide sequence identity could be as low as 82.5%, when local and commercial vaccine isolates were compared (range, 82.5-93.8%). Additionally, since Vacc-P was genetically distinct from other CDV vaccine strains, the lineages origin of Vacc-P strain is necessary to be clarified. Based on the sequence alignment of full length F gene, Vacc-P has its nucleotide identity as high as 99.3% when comparing with Vaccine X strain (GenBank: EU072198) (Data not shown), which clearly manifested that Vacc-P strain might be derived from the contemporary CDV vaccine strain as Vaccine X. These findings indicated that the sequence variation of CDV circulating in Taiwan and the currently used commercial vaccines is significant. Also, the contemporary distemper vaccines, such as Vacc-P and Vaccine X, are commonly used in Taiwan.

**Table 1:** Comparison of the nucleotide sequences of field isolates from Taiwan with commercial CDV vaccines using the CDV M-F UTR and part of the F gene (nucleotides 4325-5325)

Percentage identity										
	TW-1	TW-2	TW-3	TW-4	TW-5	TW-6	TW-7	Vacc N	Vacc P	Vacc Q
TW-1		98.5	99.3	98.1	97.6	98.0	98.1	83.3	93.6	85.1
TW-2	1.5		98.6	97.8	97.9	97.7	97.8	82.7	93.8	85.0
TW-3	0.7	1.4		98.2	97.7	98.1	98.2	82.9	93.5	84.8
TW-4	1.9	2.2	1.8		96.9	99.9	100.0	83.1	93.5	84.9
TW-5	2.4	2.1	2.3	3.2		96.8	96.9	82.5	93.2	85.0
TW-6	2.0	2.3	1.9	0.1	3.3		99.9	83.0	93.4	84.8
TW-7	1.9	2.2	1.8	0.0	3.2	0.1		83.1	93.5	84.9
Vacc N	19.3	20.2	19.9	19.6	20.4	19.7	19.6		86.2	96.3
Vacc P	6.7	6.5	6.8	6.8	7.2	7.0	6.8	15.6		88.9
Vacc Q	16.9	17.1	17.3	17.2	17.0	17.3	17.2	3.8	12.2	
**Divergence**										

**Figure 2:**
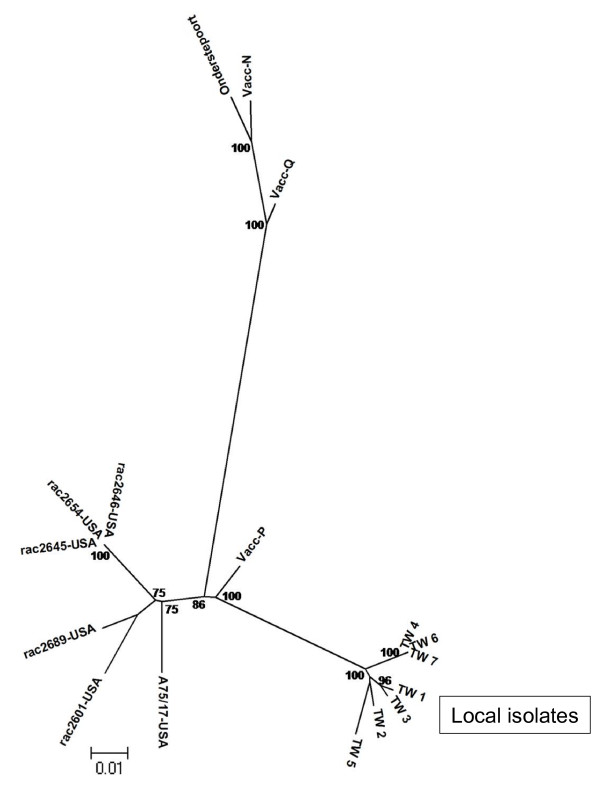
**Phylogenetic analysis of various CDV strains based on the nucleotide sequence of part of the F protein and the intergenic region between the M gene and the F gene (nucleotides 4325-5325)**. Only bootstrap values greater than 70 are shown and the branch lengths are proportional to genetic distance.

**Figure 3:**
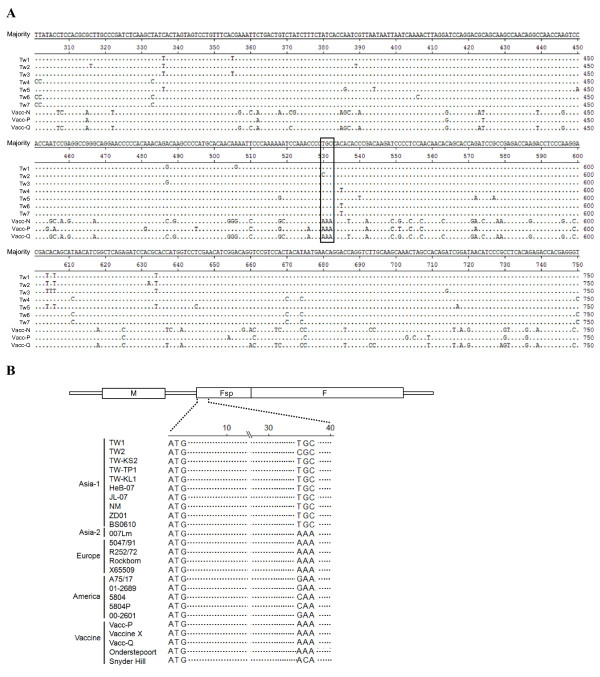
**Sequence alignment of partial F gene**. (A)The F gene nucleotide sequences, including the intergenic region between M and F gene (M-F UTR), of field strains from Taiwan and three commercial vaccine strains were analysed. The numbering starts at the first amino acid of the M-F UTR. Only amino acids that differ from the majority sequence are shown. Identical residues are represented by dots. The substitution of the AAA present in the vaccine strains, which was used to design the differentiating primers for ARMS-PCR, is indicated by a square box. (B) The region consisting of TCG motif, located 35 nucleotide downstream from the start codon (ATG) was comparatively aligned with various CDV lineages: Asia-1 strain; TW-KS2, TW-TP1, TW-KL1, HeB-07, JL-07, NM, ZD01, BS0610 (GenBank: EU192013, EU191985, EU191988, EU327874, EU327875, EF596903, EF596904, EU934234), Asia-2 strain; 007 Lm (GenBank: AB474397), Europe strain; 5047/91, R252/72, Rockborn, X65509 (GenBank: AF026240, AF026243, AF026244, X65509), America strain; A75/17, 01-2689, 5804, 5804P, 00-2601 (GenBank: AF164967, AY649416, AY386315, AY386 316, AY443350), and Vaccine strain; Vaccine X, Snyder Hill and Onderstepoort (GenBank: EU072198, GU138403, AF305419). Omitted sequences are represented by dots.

Phylogenetic analysis of these nucleotide sequences in conjunction with CDV strains from other continents available in the GenBank database was then carried out. The phylogenetic tree, as shown in Fig. 2, demonstrated that all local isolates formed a single clade, which was distant from the vaccine isolates and other field isolates from America. Among the vaccine strains, Vacc-P, the contemporary vaccine strain, was freestanding and located between our local isolates and the CDV strains isolated in America. This contrasted with Vacc-N and Vacc-Q, which were clustered in the same group as the Onderstepoort vaccine strain. A bootstrap value of 100 for this clade suggests a robust phylogenetic grouping. Noticeably, the sequence variation events among the local isolates and the commercial vaccines observed in the M-F intergenic region and the pre-signal peptide region of F gene were well scattered (Fig. 3A).

If these results are examined as a whole, all of the local isolates were found to be closely related to strains belonging to the Asia-1 lineage, which is distant and phylogenetically distinct from the vaccine strains. Additionally, the analysis of the three commercial vaccines indicated that two out of the three seem to have originated from a common ancestor similar to other vaccine strains (Onderstepoort and Convac), while only Vacc-P strain has a closer phylogenetic relationship with our local strains.

### Differentiation of the vaccine strains and the field CDV isolates by Multiplex ARMS-PCR

Amplification refractory mutation system (ARMS)-PCR, also called allele-specific oligonucleotide PCR, was originally designed for the detection of known sequence polymorphisms, such as point mutations [24]. Using just two pairs of primers in a single PCR tube, this method can simultaneously amplify both mutant and wild type alleles, plus it allows for the amplification of an internal DNA control. This technique has been applied to the genotyping, analysis of genetic disorders [25-27], and the diagnosis of several different virus infections [26,28,29]. The discrimination of amplification mainly depends on the mismatch nucleotide at the most 3'-terminus of primer [24]. The allele-specific (or lineage-specific) priming of the PCR process will only permit amplification to occur when the most 3'-terminal nucleotide matches with its target sequences (Fig 1).

Alignment of the sequences revealed the substitution of three adenines at positions 530-532 in all three vaccine strains; while the sequences at the same positions in the local isolates are T/CGC (marked with square in Fig. 3A). Interestingly, this T/CGC, located 35 nucleotide downstream from Fsp start codon, also can be observed in other Asia-1 CDVs, including strains from Taiwan (49 strains) and China, published in GenBank database (Fig. 3B). This apparent variation allowed the design of a genotype-specific primer that would differentiate local strains from the vaccine strains. With this in mind, in order to increase the discrimination power, the last three nucleotides at the 3'-end of the forward F-wt and the reverse R-vacc primers were designed to specifically target this particular region of the wild type or field isolates, respectively. In addition, two universal outer primers, reverse R-wt and forward F-vacc were designed to act as primer pairs for nested ARMS-PCR amplification (Fig. 1).

**Figure 4:**
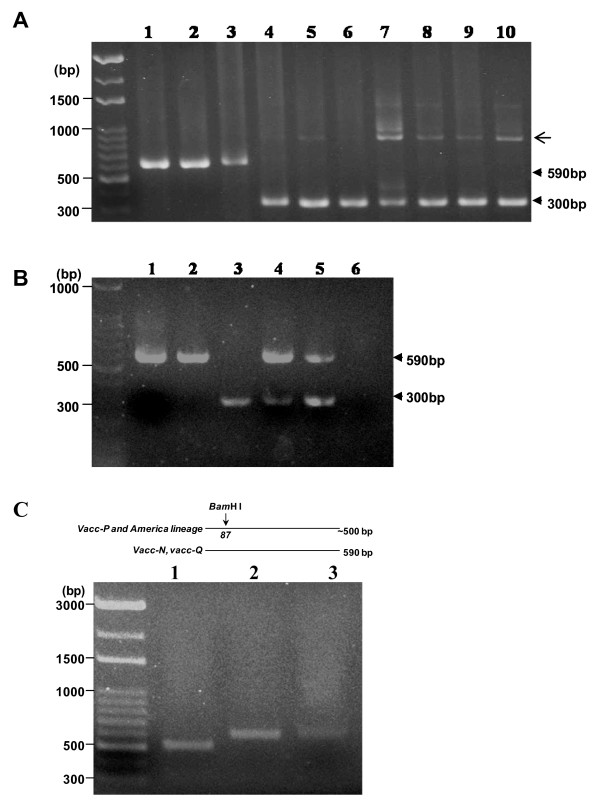
**Differential diagnosis of natural canine distemper virus infection by multiplex ARMS-PCR**. (A) Results of a multiplex PCR using the two primer pairs: F-vacc/R-vacc and F-wt/R-wt. As indicated by the arrowheads, a 590 bp product corresponding to vaccine tempalte was specifically amplified from cDNA of Vacc-P, Vacc-Q and Vacc-N (lane 1 to 3); the 300 bp product was only amplified from the local strains (lanes 4-10). Note: Bands with a higher molecular weight, indicated with an arrow, were products amplifed by the outer primer set, F-vacc and R-wt. (B) Characterisation of CDV strains by the two sets of genotype specific primers in combination with various templates, namely Vacc-P (lane 1), Vacc-Q (lane 2), a local strain (lane 3), Vacc-P and a local strain (lane 4), Vacc-Q and a local strain (lane 5) and a negative control without template (lane 6). As indicated with arrowheads, the amplicons corresponding to a specific template, the vaccine strains (590 bp) and the local strains (300 bp), can be differentiated. (C) RFLP analysis of CDV vaccine strains. A unique *Bam*H I recognition site was found in Vacc-P and CDV isolates in America lineage, but not in Vacc-N, Vacc-Q and other CDVs in Vaccine lineage. As shown in the lower panel, digestion of Vacc-P PCR product with *Bam*H I enzyme resulted in a smaller DNA fragment (~500 bp; lane 1), whereas DNA obtained from Vacc-Q and Vacc-N remained intact (590 bp; lane 2 and 3).

The region made up of nucleotides 4325-5325, which corresponds to part of the M gene, the intergenic spacer between the M and F genes and part of the F gene, was initially synthesised from the cDNAs of the seven CDV field isolates and the three commercial vaccines using the primer set CDF-F and CDF-R. The resulting amplicons were subsequently amplified using the two type specific primers sets, F-vacc/R-vacc and F-wt/R-wt (Fig. 1). The second-round PCR products represent the various genetic clusters. As illustrated in Fig. 4A, all commercial vaccine isolates were recognised by the primers F-vacc and R-vacc and yielded products that were 590 bp in length, while all seven local isolates yielded 300 bp-products when amplified by primers F-wt and R-wt.

Moreover, in order to further evaluate whether this multiplex assay can be used to characterise vaccine strains among the local strains, we performed PCR with the two sets of primers and different combinations of templates, such as one of the vaccine strains with or without the presence of a field isolate. The results consistently produced the correct 590 bp and 300 bp PCR products according to the templates present in the amplification (Fig. 4B). No cross-reactivity between the heterotypic primer pairs and the CDV strains was observed, this indicates that the multiplex ARMS-PCR is able to distinguish local isolates from vaccine strains even in a mixed population.

Base on the sequence homogeneity in agreement with the vaccine lineage, within the similar position of 590bp-product in other CDV strains from GenBank, three adenosines (AAA) located 35 nucleotide downstream from Fsp start codon, were observed in CDV isolates from Asia-2, America, Europe lineages (Fig. 3B), indicating that the forward F-vacc and reverse R-vacc primers, designed to circumstantially target AAA motif (Fig 1), are potentially able to amplify 590bp-products for recognition of these three CDV lineages. Thus, in order to specify these CDVs from vaccine strain, the further genotyping assay is needed to develop.

### Genotyping of CDV vaccine strains by restriction fragment length polymorphism (RFLP)

Within 590 nucleotides, the recognition site of *Bam*H I was observed in contemporary vaccine, but not in CDV-Vaccine cluster. The RFLP analysis was performed to differentiate contemporary vaccine from other vaccine strains. As expected, a smaller fragment of 504 bp was detected from Vacc-P amplicon digested with *Bam*H I, whereas the other two vaccines remained undigested (Fig. 4C). Thus, these results indicated that the RFLP analysis may be applied for further characterized the contemporary vaccine strain from other vaccine strains.

## Discussion

In this study, differential ARMS-PCR and RFLP genotyping system were established on the basis of the genetic divergence spanning from the intergenic region of the M and F genes to the Fsp region of F gene. The level of genetic variation of the F gene between the vaccine and circulating CDV strains in Taiwan was documented in our previous study [22]. Here we showed that, in addition to the F gene, low nucleotide similarity was found across the intergenic region of the M and F genes between the vaccine and field strains. Our results are consistent with a previous report, in which the genetic divergence of the M-F UTR was approximately two-fold higher than that of the most divergent coding sequence of the H gene [30].

Interference due to the presence of pre-existing antibodies produced by vaccination or a previous infection will affect the results of any serological diagnosis of CDV. In order to reinforce the interpretation resulting from serology based methods, the development of a method that allows the diagnosis and differentiation of CDV acquired by natural infection from that used for vaccination is worthwhile. Martella et al (2007) developed an RT-PCR genotyping system based on the lineage-specific nucleotide polymorphisms scattered over the H gene. Their system was used to characterise the major CDV lineages; European, Asia-1, Asia-2, Arctic, and Vaccine strains [12]. However, because of limitations in primer design, this system was not able to amplify CDV belonging to the America cluster and vaccine X. Very recently, another multiplex PCR assays was reported by Si et al (2010); in which primers targeting H gene was designed to distinguish field strains from China and strains from vaccine cluster, i.e. Onderstepoort [21]. Likewise, Uema et al (2005) reported that presence of *Eco*RV and *Ssp *I enzyme recognition sites of H gene in Asia strains was able to differentiate those without these sequences i.e. vaccine strains [14]. However, within the same DNA fragment, the *Ssp *I site also found in Vacc-P and American CDVs (Data not shown), indicating that this method was not able to differentiate contemporary vaccine strains from CDVs in Asia-1 and Asia-2 lineages. Therefore, the PCR genotypic system and RFLP assay targeting on H gene described previously will be jeopardized when the vaccine derived from contemporary virus strain were generally conducted.

In this study, the highly conserved TGC at positions 530-532 in pre-signal peptide (Fsp) of the local strains (Fig. 3A) allowed us to design genotype specific primer pairs to distinguish local CDV strains (Asia-1) from three vaccines, including the contemporary strains (Fig. 1). As expected, the size difference between the vaccine specific and field strain specific products provided a simple and reliable method of identification and differentiation of CDV (Fig. 4A), even when mixed templates from field and vaccine strains were used (Fig. 4B). Although the identity of the Fsp amino acid sequence, when the Taiwan strains and vaccine strains are compared, was as low as 64-67% [22], surprisingly, an analysis of Fsp region in various CDV strains in GenBank database indicates that TGC motif used to specifically target local isolates is highly conserved among the Asia-1 lineage. These findings demonstrated that our assay will be able to reliably differentiate field CDV (Asia-1 lineage, as tested in present study) from the two major lineages of conventional vaccines, namely Vacc-N/Vacc-Q and contemporary vaccine, namely Vacc-P.

In addition to differential diagnosis of natural CDV infection, the highly genetic variation of M-F UTR throughout 590 nucleotides allowed us to design the RFLP genotyping system based on the unique restriction enzyme profile. In this region, the recognition site of *Bam*H I was observed in contemporary vaccine, America, and Asia-2 clusters, but not in CDV-Vaccine cluster. Furthermore, the restriction patterns of enzyme *Apo *I and *Bgl *I were different among contemporary vaccine, America and Asia-2 lineages (Table 2). Taking together, the RFLP assay with these restriction enzymes could be potentially used in for genotyping those CDV lineages that will be useful for identification of CDV infection acquired from other lineage and also for monitoring the evolution of CDV viruses. Notwithstanding, due to the limitation of clinical specimens from other geographic areas, we was able to affirm the differentiation of circulating CDV-Asia1 strains from vaccines and contemporary vaccine.

**Table 2:** Comparison of the restriction enzyme profile within 590 nucleotide of non-coding region between M and F gene (nucleotide 4403-4492) in different CDV lineages.

lineages	Isolates	Restriction enzyme recognition site			Expected size of fragments (base pair)
		
		*Bam*H I	*Bgl *I	*Apo *I	
Vaccine	Onderstepoort Synder Hill Vacc-Q Vacc-N	-	-	**+**	27, 263

Contemporary vaccine	Vacc-P	**+**	**+**	**+**	27, 40, 19, 504

America	00-2601 00-2689 98-2645 98-2646 98-2654	**+**	**-**	**++**	27, 59, 196, 308

Asia-2	007 Lm	**+**	**-**	**-**	27, 59, 504

## Conclusions

At present, control of CDV relies on immunisation with vaccines, mostly live attenuated vaccines. A multiplex ARMS-PCR assay developed in this study can be considered as a practical and robust tool for the rapid differentiation of current circulating CDV and vaccine strains based on the sequence polymorphism in the F gene and its upstream M-F UTR. When used clinically, this assay, for the first time, is able to effectively identify the origin of a CDV infection and, most importantly, confirm the presence of a natural CDV infection.

## Methods

### Sample collection and preparation

Regardless of vaccination history, seven isolates of CDV were obtained from dogs' nasal swabs with the clinical suspicion of canine distemper provided by the Veterinary Teaching Hospital of National Chung Hsing University and by the Taichung City Animal Protection and Health Inspection Center. Nasal swabs were homogenised in 1 ml of phosphate buffered saline (PBS) and then centrifuged at 8,000 *g *for 1 min. Supernatants were collected and kept at -80°C for further experiments. In addition, three live-attenuated commercial vaccines, Vacc-P, Vacc-N and Vacc-Q, all currently used in Taiwan, were also included in this study.

### Purification of the nucleic acid, reverse transcription and amplification of F gene

Total nucleic acid was extracted from the supernatants of swabs and vaccines using the RNeasy Mini 50 kit (QIAGEN) according to the manufacturer's instructions. Total RNA (1 μg) and random 8-mer primers (50 μM) were denatured at 65°C for 5 min and cooled down on ice. To synthesise the first-strand cDNA, the RNA and primers were mixed in 5 × reaction buffers, 0.1 M DDT, 0.5 mM of each deoxynucleotide, 200 U SuperScript III reverse transcriptase (Invitrogen) and 40 U RNase inhibitor. A total of 20 μL of the mixture was initially incubated at 25°C, then the reaction was held at 65°C for 60 min and finally it was terminated by incubation at 70°C for 10 min. Following this, the first round amplification was conducted by polymerase chain reaction (PCR) with the outer primers CDF-F: 5'-AGAGTGCAAAATAGTAAGAATCCAAGC-3' and CDF-R: 5'-GAAAGAGACTGGCTATTCCGATGC-3', which amplified a fragment containing the M gene (115 downstream nucleotides; 4325-4439), M-F UTR (495 nucleotides; 4440-4934) and the F gene (first 391 nucleotides; 4935-5325) (Fig. 1). Thermocycling conditions for amplification started with an initial denaturation at 95°C for 5 min and then the reaction mixture was subjected to 35 cycles of heat denaturation at 95°C for 1 min, primer annealing at 55°C for 2 min, DNA extension at 72°C for 2 min; this was followed by a final extension at 72°C for 7 min. The identity of the resulting PCR products was verified by direct automated sequencing.

### The Multiplex ARMS-PCR assay

The F gene products from the first round PCR were then further simultaneously amplified by multiplex ARMS-PCR using two primer sets in order to distinguish the vaccine and field strains. The specific primer sets, namely F-wt and R-vacc, were designed according to the different sequences obtained and specifically targeted either the field isolates or the vaccine strains (Fig. 1). The primers used for vaccine strain amplification were F-vacc: 5'- CATCAGCCATGATCAGGGTCTTTTC-3' and R-vacc: 5'-GGGCGGTCTTGTTGGGTATGTGTTT-3'. The primers used for field strain amplification were F-wt: 5'-AATTCCCAAAAAATCCAAACCCTGC-3' and R-wt: 5'-GATTGCCGCCTCTTGAACCAGGAA-3'. The amplification conditions for the multiplex-nested ARMS-PCR were 95°C for 5 min followed by 35 cycles of denaturation at 95°C for 1 min, annealing at 55°C for 2 min, DNA extension at 72°C for 2 min and a final extension at 72°C for 7 min. All amplification cycles were performed in a DNA thermal cycle (GeneAmp PCR system 2700). The PCR products were resolved by 1.2% agarose gel electrophoresis with Health safe nucleic acid stain. Product sizes were determined with reference to a 100 base pair (bp) DNA Ladder.

### Restriction Fragment Length Polymorphism (RFLP) analysis

For genotyping, the PCR product amplified with primers F-vacc and R-vacc was isolated by using the Purelink™ PCR purification kit (Invitrogen), and resulting product was further digested with restriction enzyme *Bam*H I (New England Biolabs). A 4 ml-aliquot was digested with 1.5 U of *Bam*H I at 37°C for 90 min according to the manufacturer's recommendation. The resulting restriction fragments were resolved by 1.2% Tris acetate-EDTA-agarose gel electrophoresis.

### Phylogenetic analysis

Several CDV strains were selected for phylogenetic analysis. The nucleotide sequence accession numbers in the GenBank database for the F gene and its upstream region, M-F UTR, sequences of the reference strains used in this study are: A75/17-USA (GenBank: AF164967), Raccoon 00-2601-USA (GenBank: AY443350), Raccoon 00-2689-USA (GenBank: AY649446), Raccoon 98-2646 (GenBank: AY542312), Raccoon 98-2654 (GenBank: AY466011), Raccoon 98-2645 (GenBank: AY445077) and Onderstepoort (GenBank: AF305419).

Nucleotide sequences corresponding to the CDV F and H genes were aligned using the CLUSTAL W multiple alignment method with BioEdit software [31] and compared with other previously published sequences reported in GenBank. The phylogeny of the nucleotide and amino acid alignments were analysed using distance matrix methods (DNADIST for nucleotide sequence and PROTDIST for amino acid sequence, followed by NEIGHBOR) using the PHYLIP software package [32]. The datasets were subjected to bootstrap analysis based on 1,000 re-samplings of the original data and the SEQBOOT program was used to produce a majority-rule consensus tree.

## Competing interests

The authors declare that they have no competing interests.

## Authors' contributions

SC conducted most of this work under supervision of W-L H and T-J C. M-S L, C-Y W, and S-S C participated in clinical sample collection. K-H L, F-Y L, and T-H H participated in the sequence analysis of H gene under supervision of M-L W. All authors have read and approved the manuscript.

## Supplementary Material

Additional file 1 **Phylogenetic analysis of CDV strains based on the deduced 331 amino acid sequence of the H protein**. Only bootstrap values greater than 70 are shown, and branch lengths are proportionate to genetic distances. The accession numbers of H gene sequences of the reference strains are: Onderstepoort (AF378705), Convac (Z35493), SnyderHill (AF259552), Yanaka (D85755), Ueno (D85753), Hamamatsu (D85754), KDK1 (AB025271), Raccoon dog-Japan (AB016776), Dog98-002 (AB025270), Dog5B (AY297453), DogHM-3 (AB040767), Dog26D (AB040766), Dog5VD (AY297454), Dog-TW (AY378091), Dog5804-Germany (AY386315), Giant Panda-China (AF178038), Dog-China (AF172411), PDV-2 Siberian seal (X84998), Dog-Turkey (AY093674), Dog91A-Denmark (AF478544), Dog91B-Denmark (AF478546), DogDen (AF478543), Dogiso-Den (AF478547), Dog Denmark (Z47761), Raccoon-USA (Z47764), Raccoon01-2689-USA (AY649446), Raccoon01-2676-USA (AY498692), Raccoon01-2690-USA (AY465925), Raccoon00-2601-USA (AY443350), Jevelina-USA (Z47765) and A75-17 (AF164967). Click here for file
