# Supplementary figures and images for: Multiplex Amplification Refractory Mutation System Polymerase Chain Reaction (ARMS-PCR) for diagnosis of natural infection with canine distemper virus
Source: Virol J. 2010 Jun 10;7:122. doi: 10.1186/1743-422X-7-122 (PMC2907576; doi:10.1186/1743-422X-7-122)

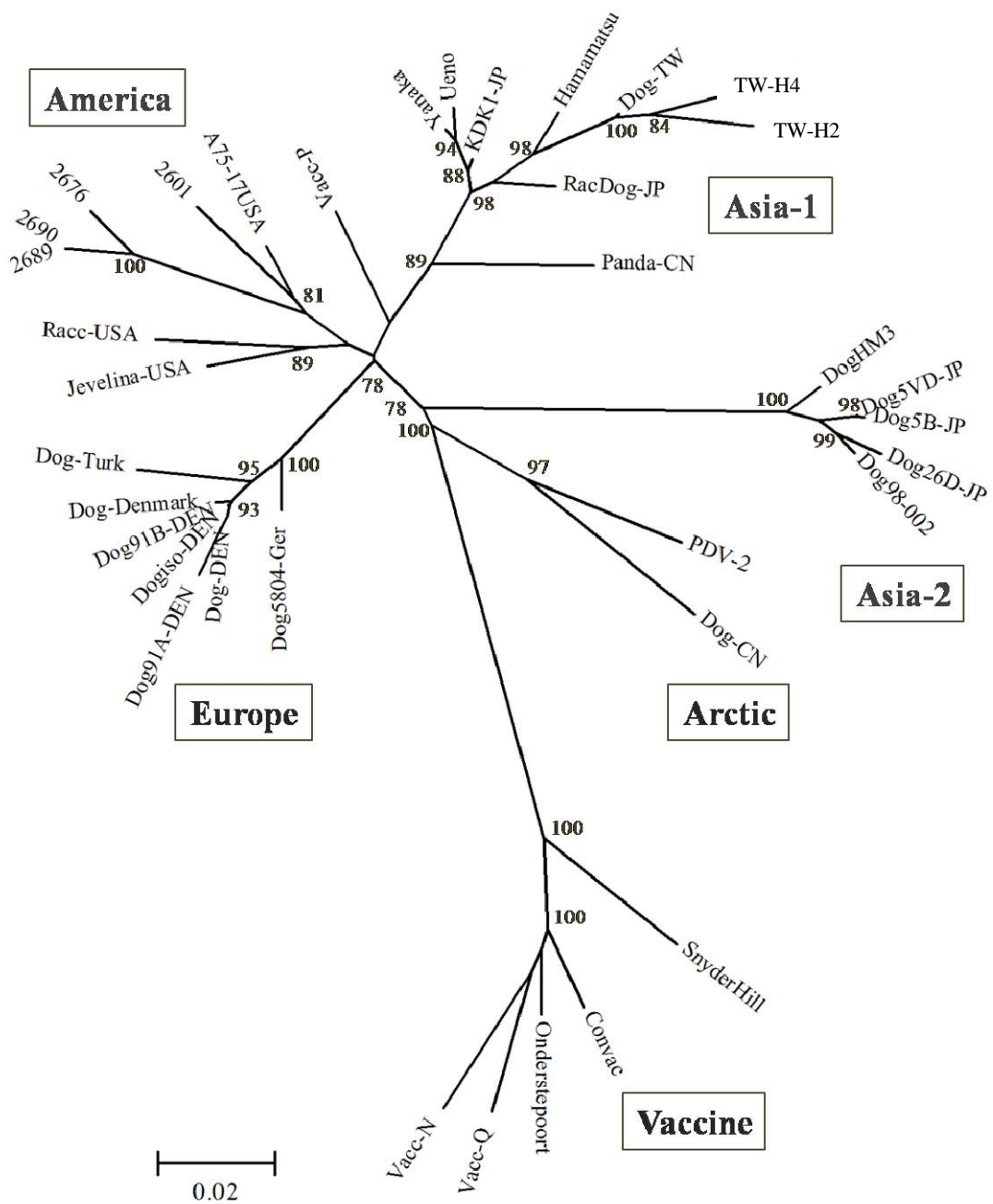

Supplement: Additional file 1 — Phylogenetic analysis of CDV strains based on the deduced 331 amino acid sequence of the H protein. Only bootstrap values greater than 70 are shown, and branch lengths are proportionate to genetic distances. The accession numbers of H gene sequences of the reference strains are: Onderstepoort (AF378705), Convac (Z35493), SnyderHill (AF259552), Yanaka (D85755), Ueno (D85753), Hamamatsu (D85754), KDK1 (AB025271), Raccoon dog-Japan (AB016776), Dog98-002 (AB025270), Dog5B (AY297453), DogHM-3 (AB040767), Dog26D (AB040766), Dog5VD (AY297454), Dog-TW (AY378091), Dog5804-Germany (AY386315), Giant Panda-China (AF178038), Dog-China (AF172411), PDV-2 Siberian seal (X84998), Dog-Turkey (AY093674), Dog91A-Denmark (AF478544), Dog91B-Denmark (AF478546), DogDen (AF478543), Dogiso-Den (AF478547), Dog Denmark (Z47761), Raccoon-USA (Z47764), Raccoon01-2689-USA (AY649446), Raccoon01-2676-USA (AY498692), Raccoon01-2690-USA (AY465925), Raccoon00-2601-USA (AY443350), Jevelina-USA (Z47765) and A75-17 (AF164967). [file 1743-422X-7-122-S1.PDF]
